# Subject-specific modelling of pneumoperitoneum: model implementation, validation and human feasibility assessment

**DOI:** 10.1007/s11548-019-01924-2

**Published:** 2019-02-20

**Authors:** Mafalda Camara, Shivali Dawda, Erik Mayer, Ara Darzi, Philip Pratt

**Affiliations:** Department of Surgery and Cancer, Imperial College London, London, UK

**Keywords:** Pneumoperitoneum, Surgical planning, Biomechanical modelling, Position-based dynamics

## Abstract

**Purpose:**

The aim of this study is to propose a model that simulates patient-specific anatomical changes resulting from pneumoperitoneum, using preoperative data as input. The framework can assist the surgeon through a real-time visualisation and interaction with the model. Such could further facilitate surgical planning preoperatively, by defining a surgical strategy, and intraoperatively to estimate port positions.

**Methods:**

The biomechanical model that simulates pneumoperitoneum was implemented within the GPU-accelerated NVIDIA FleX position-based dynamics framework. Datasets of multiple porcine subjects before and after abdominal insufflation were used to generate, calibrate and validate the model. The feasibility of modelling pneumoperitoneum in human subjects was assessed by comparing distances between specific landmarks from a patient abdominal wall, to the same landmark measurements on the simulated model.

**Results:**

The calibration of simulation parameters resulted in a successful estimation of an optimal set parameters. A correspondence between the simulation pressure parameter and the experimental insufflation pressure was determined. The simulation of pneumoperitoneum in a porcine subject resulted in a mean Hausdorff distance error of 5–6 mm. Feasibility of modelling pneumoperitoneum in humans was successfully demonstrated.

**Conclusion:**

Simulation of pneumoperitoneum provides an accurate subject-specific 3D model of the inflated abdomen, which is a more realistic representation of the intraoperative scenario when compared to preoperative imaging alone. The simulation results in a stable and interactive framework that performs in real time, and supports patient-specific data, which can assist in surgical planning.

## Purpose

In laparoscopic procedures, the abdominal cavity is inflated with $\hbox {CO}_{2}$ to a pressure between typically 10 and 16 mmHg, creating pneumoperitoneum. This insufflation induced between the internal organs and abdominal wall provides a workspace which allows triangulation of surgical instruments and the surgical viewpoint entry necessary to perform the procedure safely [1]. Laparoscopy is a complicated procedure, and significant research has been performed to assist the surgeon in the fields of intraoperative navigation and surgical planning. These are most commonly driven by preoperative 3D imaging (e.g. CT and MRI) and aim to simplify the procedure. However, intraoperatively the organs of the abdominal cavity deform and move significantly due to surgical manipulation, pneumoperitoneum, breathing and vessel pulsation. The pneumoperitoneum alone is estimated to induce a displacement to or movement of internal organs and the abdominal wall of up to 4 cm [2, 3]. These intraoperative phenomena change the anatomy with respect to preoperative imaging, thereby making the latter not a reliable prediction of the intraoperative environment. Therefore, using preoperative imaging without accounting for such changes is potentially misleading for the surgeon.

In laparoscopy, port placement through the abdominal wall enables internal access for surgical instruments and the endoscope. Optimal port placement can assist in avoiding undesired poor dexterous control over instruments, which might prevent the ability to reach the desired targets. The placement of the endoscope, which subsequently guides the placement of following trocars, is blind in the Veress entry approach. Such is a cause of complications during laparoscopy that may result in vascular or organ damage [4]. An adequate estimation of trocar positioning is therefore fundamental to ensure the ease, quality and safety of the surgical procedure. However, to date there is no standard consensus on trocar positioning across different procedures [5]. Simple and generic guidelines have been proposed in the literature [4, 5], but trocar placement is performed typically according to the surgeon’s intuition based on surgical experience. The surgeon most commonly estimates the first trocar positioning based on the patient’s preoperative images and by mentally mapping that information onto the patient under insufflation intraoperatively. Preoperative data do not consider pneumoperitoneum and cannot account for modifications that occur due to it intraoperatively. This disparity between preoperative and intraoperative imaging strengthens the need for modelling the changes that occur due to insufflation.

Bano et al. [6] developed a patient-specific model of pneumoperitoneum for surgical planning, by simulating the movement of the abdominal wall, viscera and arteries (epigastric). The authors implemented a finite element method (FEM) approach with defined biomechanical parameters on segmented anatomical structures from CT scans. The simulation was validated with porcine subjects, resulting in an accurate estimation of insufflation. However, only two datasets were used for a single pressure and no assessment on humans was performed. Kitasaka et al. [7] implemented a model to simulate and visualise a virtual pneumoperitoneum, using an elastic deformation model based on a node–spring system. The authors used a preoperative tomographic image directly as input data and the method outputted an estimate of the deformed image resulting from the simulation of pneumoperitoneum. However, the model was too time-consuming to prepare and the overall method still required validation. The same group later developed a different approach based on tracking the movement of specific landmarks intraoperatively on a patient’s abdominal wall to assess the accuracy of simulating pneumoperitoneum [8]. By measuring the landmarks’ displacements and using them to feed an optimisation of the simulation parameters, the authors achieved improved accuracy when compared to the previous experience. More recently, the same group had developed a mass–spring system (MSS) approach to estimate the geometry of pneumoperitoneum [1]. The method required as input a given pressure, known mechanical properties and tomographic data. This implementation delivered an accurate deformed tomographic image, but required around 2.5 h per dataset and for an applied pressure of 30 mmHg.

The study proposed herein implements a framework to simulate the patient-specific anatomical changes resulting from pneumoperitoneum during laparoscopic procedures, using preoperative imaging as input data. The framework combines the use of a biomechanical model that has been previously implemented and validated for a patient-specific soft tissue deformation and is described in Camara et al. [9].

The innovative aspect of this approach is the combination of the following components: a patient-specific framework that models pneumoperitoneum; the calibration of the model using multiple porcine datasets and successful validation using a porcine dataset under different levels of insufflation; a real-time interaction with the model; and the assessment of the feasibility of modelling pneumoperitoneum in humans. Furthermore, this simulation was implemented within a framework developed with a position-based dynamics approach [9], which facilitates data preparation. This model can potentially facilitate surgeons to plan the procedure preoperatively and define an optimal surgical strategy, by comprehending the patient’s anatomy under insufflation. This model could further assist the surgeon to estimate trocar positions for instruments accessibility and an optimal surgical field of view (FOV). This post-insufflation model could be additionally helpful for surgical simulators or augmented reality (AR).

## Methods

Two different groups of porcine datasets were required to calibrate and subsequently validate the pneumoperitoneum model. Group (#1) consisted of pairs of datasets from eight different porcine subjects, each pair corresponding to a pre- and a post-insufflation CT scan.^1^ Group (#2) consisted of five pairs of pre- and post-insufflation CT scans from a single porcine subject. Both groups #1 and #2 were acquired under different protocols and at different times. Datasets of group #1 consisted of full abdominal scans acquired at a pressure roughly of 12mmHg. (There was no certainty of the experimental pressure applied or observed, as the data were acquired at an external facility by a separate team; similar was assumed by Johnsen et al. [10].)^2^ These datasets were used to generate the model and calibrate the simulation parameters. Datasets of group #2 consisted of abdominal scans acquired with no pressure (pre-insufflation), under 4, 8, 12 and 16 mmHg of abdominal pressure. Datasets from this group were used to calibrate the simulation pressure parameter to the experimental pressure and validate the model.

### Data preparation

3D models were generated from the datasets of inflated and deflated scans. Deflated models were imported into the simulation environment and subjected to different pressure levels to create a virtual pneumoperitoneum. These simulated models were compared to the ground truth reference models, which corresponded to the insufflated CT scans.

***Dataset # 1*** Eight porcine subjects were inflated to a pressure roughly of 12mmHg. Contrast-enhanced CT scans were acquired with a GE LightSpeed16, when the subject was inflated and repeated after deflation, with subjects laying in a supine position. Acquisitions resulted in a pair of datasets per subject: an inflated and a deflated volume (resolution of $0.85\times 0.85\times 2.5$ mm). Each volume was partially segmented manually using ITK-SNAP 3.6.0 [11] into three structures: the viscera (vasculature, muscles, fat and abdominal visceral organs), the abdominal wall (skin, fat, muscle, the spine and ribs) and pneumoperitoneum (defined as the boundary between the abdominal wall and the viscera). Each of the segmented structures was imported as a surface mesh to MeshLab 1.3.3 [12], underwent quadric edge collapse decimation (quality threshold 0.99) [13] and was smoothed using the volume-preserving Humphrey’s classes (HC) Laplacian algorithm [14]. All of the resulting meshes were scaled down to half of the original size to ensure real-time performance and to maintain an appropriate particle radius. Such is comparable to increasing the particle radius, but corresponds to a more straightforward implementation.

***Dataset # 2*** A single subject was CT-scanned in a supine position (resolution of $0.45\times 0.45\times 0.45$ mm) for increasing levels of abdominal pressure, starting with no pressure and under 4, 8, 12 and 16mmHg. The volumes were partially segmented automatically using the *F.A.S.T.*^®^*Interactive Segmentation*^3^ [15], to ensure an accelerated segmentation process, and imported later to ITK-SNAP for manual adjustments. The meshing steps for these datasets were the same steps used in group #1.

### Implementation

**Fig. 1:**
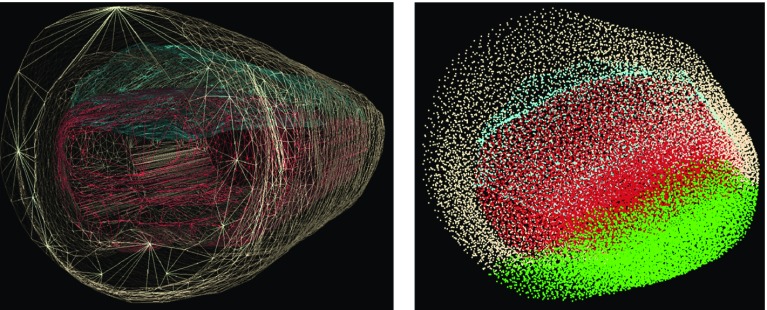
Left: surface meshes of the abdominal wall (pink), viscera (red) and pneumoperitoneum (blue) used in the ‘skinning’ technique. Right: particle distribution of the same structures; green represents fixed particles that correspond to the region of the back

The simulation was implemented within the GPU-accelerated NVIDIA FleX position-based dynamics framework [16], in a manner similar to that reported in Camara et al. [9]. The FleX engine supports different modelling structures and collision geometries. The abdominal wall and viscera were modelled as soft bodies: the surface meshes were discretised into particles and grouped together into clusters. The deflated pneumoperitoneum was modelled as an inflatable structure. Inflatables are modelled by combining a cloth mesh with a global volume constraint, as described in Macklin et al. [17]. An additional spring stiffness constraint parameter was used to control the stiffness of the inflatable. To model pneumoperitoneum, the deflated surface mesh was used to generate the surface cloth and a factor variable, the global volume constraint (herein named ‘simulation pressure parameter’) varied to model the different levels of applied pressure. This parameter controls the user-defined volume of the model as a percentage of original volume. Figure 1 represents the modelled structures under the FleX engine.

Particles that were positioned within a predefined distance (centre of mass of the viscera minus 20 mm on the y-axis) were fixed (left image of Fig. 1). Acceleration due to gravity was set to zero in all directions for simplification, as the simulation parameters undergoing calibration were not yet estimated. A gravity compensation should be performed ideally and discrepancies should be in the order of the sub-millimetre [9]. A ‘skinning’ technique [18] was used with the surface meshes of all the structures modelled: pneumoperitoneum, abdominal wall and viscera (right image of Figure 1). This ensured that the surface geometries attached to the respective system of particles would deform in accordance with the manipulation of the same bodies.

### Calibration with porcine dataset #1

A simple four-dimensional exhaustive search was performed to establish the optimal set of simulation parameters. The metric undergoing minimisation was defined as the average of the nearest distance between the vertices of the simulated inflatable and the vertices of the reference pneumoperitoneum from the inflated CT scan. The optimisation was performed on each porcine dataset (#1) and compared across the entire group. The set of parameters that minimised the error metric over all porcine subjects was the set adopted for the validation of the model. The parameters undergoing calibration are defined in Table 1. The range adopted for each parameter was based on previous experience. The remaining simulation parameters were defined as in Camara et al. [9].

**Table 1:** Simulation parameters used for model calibration

Parameter	Range
Cluster stiffness	0.4, 0.5, 0.6, 0.7, 0.8
Spring stiffness	[0.1, 1.0], with increments of 0.1
Particle radius (mm)	2.2, 2.7, 3.3
‘Simulation pressure parameter’	[1.0, 15.0], with increments of 0.5

All parameters are dimensionless, aside from the particle radius

**Fig. 2:**
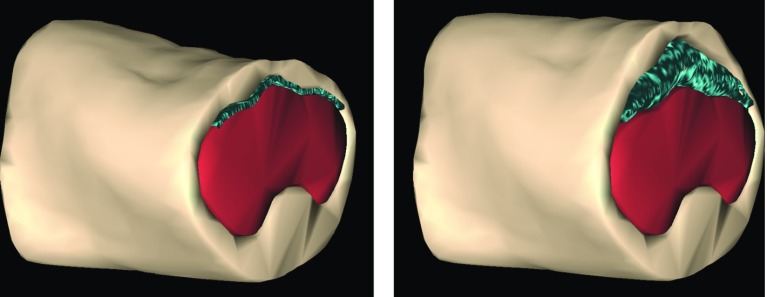
Model before (left) and after (right) insufflation, with a corresponding expansion of the abdominal wall (pink) and compression of the viscera (red). In this example, an increase of 10$\times$ in the inflatable (blue) original volume resulted of setting the simulation pressure parameter to 10

The cluster stiffness characterised the stiffness of the viscera and abdominal wall soft bodies; the spring stiffness defined the stiffness of the inflatable structure; the particle radius defined the density of the particle distribution; the ‘simulation pressure parameter’ determined the multiplication factor in the original volume of the cloth mesh used to generate the inflatable structure and does not correspond directly to the experimental abdominal pressure. The correspondence between this ‘simulation pressure parameter’ and the experimental insufflation pressure is addressed further in Sect. 2.4. Figure 2 illustrates the model given a variation in the ‘simulation pressure parameter’.

Once the optimal cluster stiffness, spring stiffness and particle radius were determined, the insufflation was modelled for each porcine subject across the different levels of simulation pressure. For each level, the ‘skinned’ surface meshes of all structures were extracted, with the respective error metric. These meshes and errors were scaled back to original size to ensure a correct comparison with the ground truth.

### Validation with porcine dataset #2

Having an optimal set of parameters permitted the determination of a mapping between the ‘simulation pressure parameter’ and the experimental insufflation pressure. Group #1 was not used for validation because the subjects were only inflated with an estimated pressure of 12 mmHg, thus not providing enough data points across different levels of pressure. As such, datasets from group #2 were used to assess what form of relationship exists between both variables.

The simulation was initiated with the given set of optimal parameters. The deflated model was subjected to an increased pressure by gradually increasing the ‘simulation pressure parameter’. For each level of applied pressure, the surface mesh ‘skinned’ to the inflatable structure was extracted. Each mesh was then compared to pneumoperitoneum meshes corresponding to the reference volumes of 4, 8, 12 and 16mmHg. This comparison was performed by generating an inflatable volume resulting from discretising the surface mesh into voxels (resolution of $1\times 1\times 1$ mm) for each given ‘skinned’ surface mesh. Each volume was then compared against the inflatable volume of the reference CT scan, by means of an error metric based on the total percentage of voxels from each volume that did not overlap with the other. An error metric value of zero would mean a perfect overlap.

### Feasibility of modelling pneumoperitoneum in humans

**Fig. 3:**
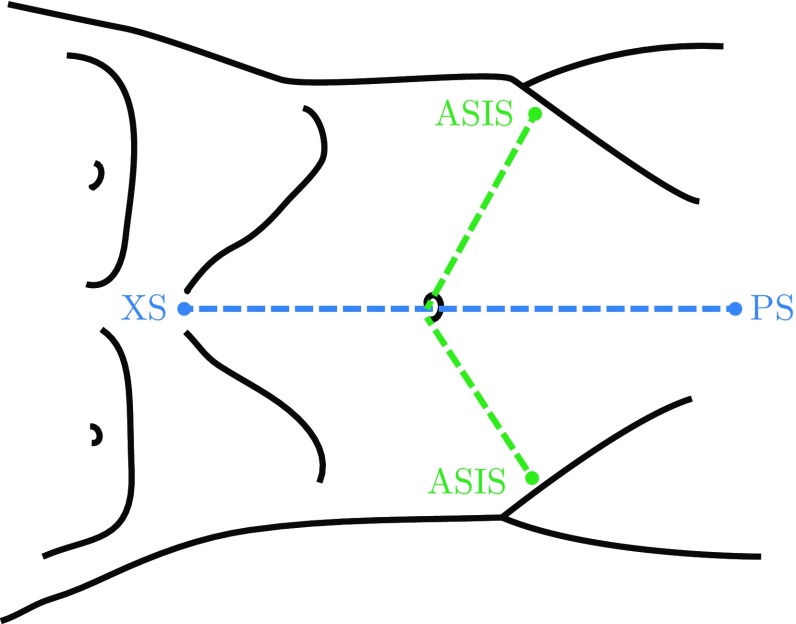
Distances measured between landmarks on the skin: xiphisternum (XS) to pubic symphysis (PS), and umbilicus to right and left anterior–superior iliac spines (ASIS)

To assess the feasibility of translating the developed model of pneumoperitoneum from porcine to human subjects, an alternative evaluation was adopted, as the approach used with porcine subjects is not feasible with patients (due to the required exposure to radiation and operating room logistics). Distance measurements between specific landmarks on the skin of the abdominal region were collected from a patient, pre- and post-insufflation intraoperatively. Concurrently, a 3D model of the patient was generated using the preoperative CT scan and used as input for the simulation. Insufflation pressure was gradually applied to the model, and the same measurements were collected before any applied pressure and again post-insufflation. This procedure resulted in pairs of measurements of pre- and post-insufflation from the actual patient and from the simulation model. The ‘simulation pressure parameter’ used was the value that matched the experimental 14 mmHg pressure used intraoperatively. The landmarks were decided given their ease to be detected in the CT scans and their accessibility to be measured physically. These landmarks are represented in Fig. 3.

The landmarks were identified and segmented from the CT scan as individual structures and alongside the abdominal wall were exported as surface meshes. The landmarks were mapped to coordinates on the abdominal wall mesh, and the distances between them were generated by computing the geodesic distance [19] with MATLAB (R2015a 8.5.0) between each pair of coordinates. Intraoperatively, the measurements were straightforward to collect using sterile drapes and later a ruler.

## Results

### Calibration with porcine data #1

**Fig. 4:**
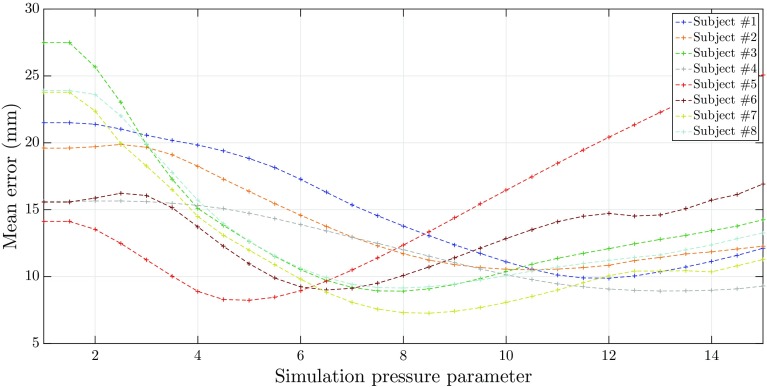
Mean error, per porcine subject, of the simulated inflatable meshes when compared to the reference meshes, as a function of the ‘simulation pressure parameter’

The set of parameters that resulted in the minimum average error is summarised in Table 2. Given this set of parameters, the average error per subject as a function of the ‘simulation pressure parameter’ is presented in Fig. 4.

**Table 2:** Optimal simulation parameter resulting from the calibration

Parameter	Optimal value
Cluster stiffness	0.6
Spring stiffness	0.5
Particle radius (mm)	2.7

The 7th porcine dataset achieved the most accurate result with a minimum mean error of 7.3 ± 2.2 mm, for a ‘simulation pressure parameter’ of 8.5. The 2nd porcine dataset achieved the least accurate result with a minimum mean error of 10.5 ± 2.8 mm, for a ‘simulation pressure parameter’ of 10.5. At the start of the simulation (no applied pressure), the mean errors were of 19.6 and 23.8 mm for the 2nd and the 7th porcine datasets, respectively. Comparing these numbers against the minimum mean error demonstrates that the difference is significant, meaning that the simulation of pneumoperitoneum resulted in an improved estimation of intraoperative scenario when compared to the preoperative model alone. Additionally, these results validated the optimisation process used. Overall, the datasets follow an intuitive trend: the mean error gradually decreased until the minimum (where the simulated inflatable was most similar to the reference pneumoperitoneum) and increased from thereafter, which illustrated an over-expansion of the inflatable over the reference pneumoperitoneum. Figure 4 demonstrates further that the minimum error occurred at different simulation pressure parameters for all subjects. This confirms the assumption that the applied pressure was indeed approximate (and estimated to 12mmHg), and as such, this dataset could not provide a proper mapping between the simulated and experimental pressures.

### Validation with porcine data #2

**Fig. 5:**
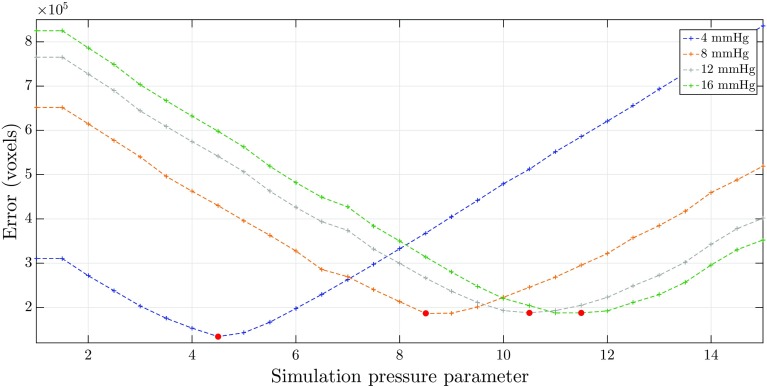
Error (count of non-overlapping voxels) of the simulated inflatable volume when compared to the reference pneumoperitoneum volume, as a function of the ‘simulation pressure parameter’. Each data series corresponds to an experimental insufflation pressure. Dots highlighted in red correspond to the minimum error found within each dataset

The error was calculated for each individual experimental pressure and generated from overlapping the simulated inflatable volume against the reference pneumoperitoneum volume. Figure 5 illustrates the errors for each experimental insufflation pressure as a function of the ‘simulation pressure parameter’.

**Fig. 6:**
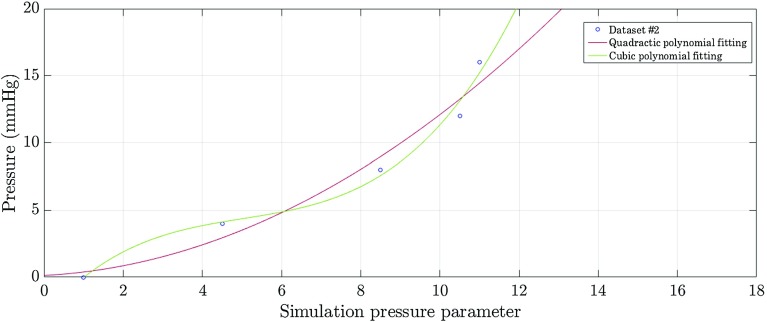
Experimental insufflation pressure as a function of the ‘simulation pressure parameter’ (blue dots). The green and red curves show the cubic and quadratic polynomial functions, respectively

The curves for 4, 8, 12 and 16 mmHg illustrate a general trend: the error decreased until the minimum (where the simulated and the reference volumes most resembled) and increased from thereafter, which illustrates the over-expansion of the inflatable in comparison with the reference pneumoperitoneum. For example, looking at the 8 mmHg dataset: the error decreased with the increase in the ‘simulation pressure parameter’ until it reached the value 8.5; here, the simulated inflatable resembled the reference pneumoperitoneum the most at 8 mmHg; beyond this point, the simulated inflatable continued to expand. Dots highlighted in red correspond to the minimum error for each dataset, where the simulated and experimental pressures best align. Using these data points, a correspondence between the ‘simulation pressure parameter’ with the respective experimental insufflation pressure is illustrated in Fig. 6. The red curve corresponds to the quadratic regression function; the green curve corresponds to the cubic polynomial regression function ($y = 0.038 x^{3} - 0.57 x^{2} + 3.4 x - 2.9$ and $R^{2} = 0.987$). This cubic regression ensures the intersection with the (1, 0) data point, which coincides with the initial configuration of the insufflation model, and provides sufficient smoothing without over-fitting. Therefore, the cubic function was chosen over the monotonically increasing quadratic fit. This regression captures the correspondence between the simulated and experimental pressures, obtained from the cubic polynomial regression.

For each experimental pressure, the ‘skinned’ meshes of the inflatable structure for each corresponding ‘simulation pressure parameter’ were extracted. The mean and maximum distances, calculated using a Hausdorff distance metric [20], between the simulated and reference pneumoperitoneum meshes, are represented in Table 3.

**Table 3:** Mean and maximum absolute distances (mm), calculated with a Hausdorff distance metric, between the simulated inflatable mesh and the reference pneumoperitoneum

Pressure (mmHg)	Mean (mm)	Max (mm)
Pneumoperitoneum, 4	5.8	19.4
Pneumoperitoneum, 8	6.2	24.4
Pneumoperitoneum, 12	5.8	23.8
Pneumoperitoneum, 16	5.3	23.5

**Fig. 7:**
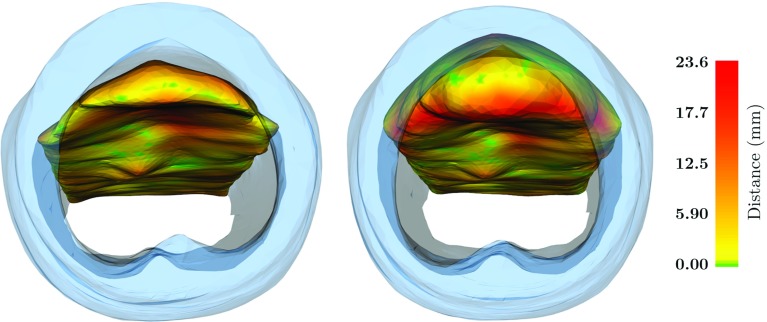
Illustration of the Hausdorff distance (mm) of the simulated inflatable against the reference pneumoperitoneum, for the 4 mmHg (left) and 16 mmHg (right) pressure datasets, through a colour map. The abdominal wall is represented in light blue

Examples of the Hausdorff distance are illustrated in Fig. 7 through a colour map. The left image shows the colour gradient for the inflatable mesh generated with a ‘simulation pressure parameter’ of 4.5 against the mesh generated from the experimental insufflation pressure of 4 mmHg. The right image shows the same but for a ‘simulation pressure parameter’ of 11.0 against an experimental pressure of 16mmHg.

### Feasibility of modelling pneumoperitoneum in humans

The human pneumoperitoneum was modelled assuming a ‘simulation pressure parameter’ of 10.5, given the experimental insufflation pressure of 14 mmHg used intraoperatively. This correspondence between pressures was retrieved from the cubic polynomial function (illustrated in Fig. 6). The distance measurements acquired from the generated model and from the patient are presented in Table 4.

**Table 4:** Measurements between landmarks in the before- and after-insufflation configurations, measured intraoperatively from the patient and generated from the simulation model

	Intraoperative (mm)			Simulation (mm)		
	$\hbox {U-ASIS}_\mathrm{R}$	$\hbox {U-ASIS}_\mathrm{L}$	XS-PS	$\hbox {U-ASIS}_\mathrm{R}$	$\hbox {U-ASIS}_\mathrm{L}$	XS-PS
Pre	180	175	328	163	169	381
Post	190	175	374	178	184	416
$\varDelta$	10	0	46	15	15	35

**Fig. 8:**
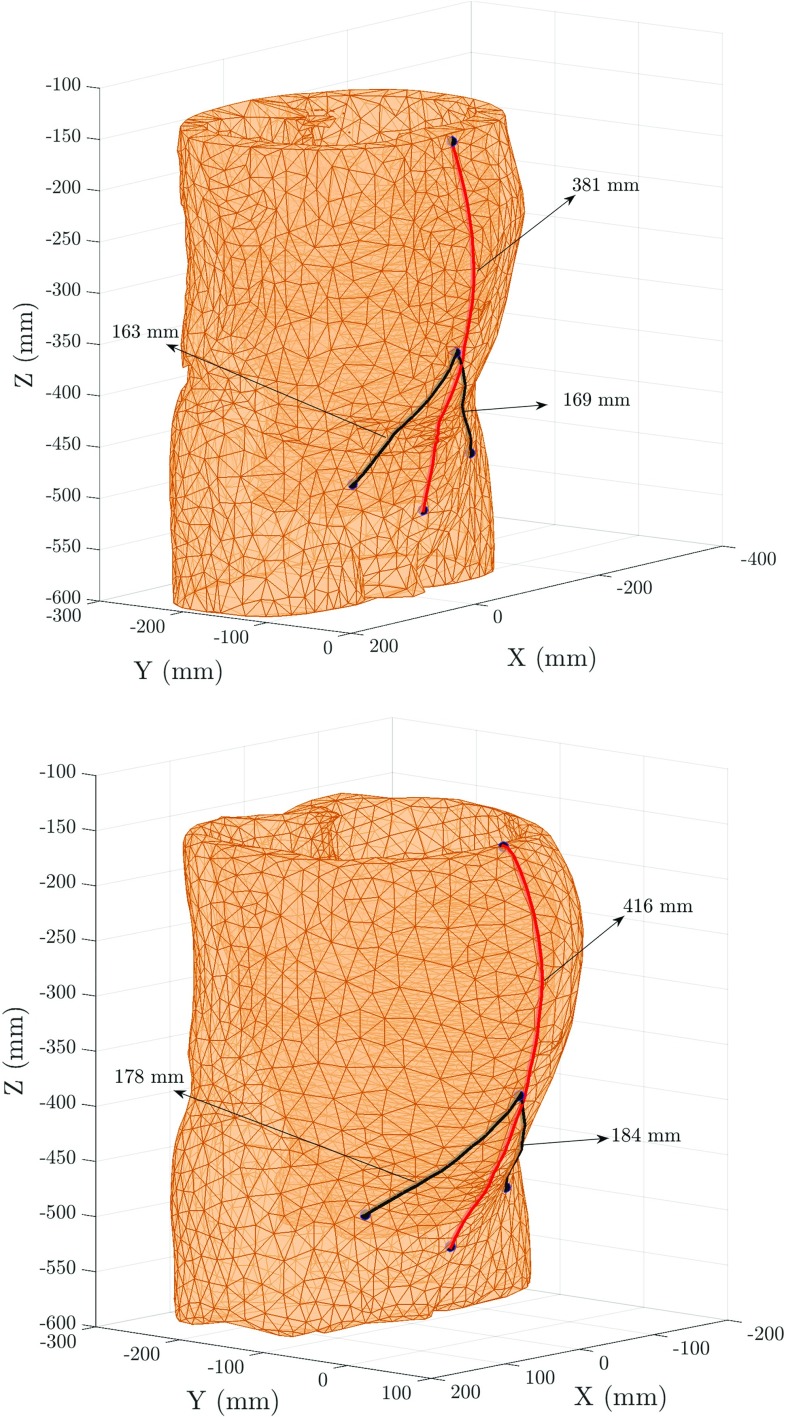
Measurements between landmarks on the pre- (top) and post-insufflation (bottom) configurations. Measurements in black correspond to the distance between the umbilicus to the right and left anterior–superior iliac spines (ASIS); measurement in red corresponds to distance between the pubic symphysis (PS) and the xiphisternum (XS)

The measurements between the different landmarks, for the pre- and post-insufflation configurations, and calculated with the geodesic distance algorithm, are illustrated in Fig. 8. Measurements in the pre-insufflation configuration were calculated with the mesh extracted from the patient CT scan directly. Measurements in the post-insufflation configuration were calculated on the abdominal wall ‘skinned’ mesh that resulted from modelling pneumoperitoneum.

## Conclusions

An accurate and realistic simulation of pneumoperitoneum using multiple porcine datasets was implemented, and feasibility of translating the insufflation model to humans was further demonstrated. Datasets from group #1 were used to generate the model and to enable a successful calibration of the simulation parameters (cluster and spring stiffness, and particle radius). Datasets from group #1 were disregarded for model validation due to the uncertainties regarding the settings used during CT acquisitions (actual applied pressure, possibility of leakages or over-insufflation). Datasets from group #2 were used to define a correspondence, given by a cubic polynomial function, between the simulation and experimental pressures. With the optimal set of parameters, the average Hausdorff distance between the simulated and reference pneumoperitoneum meshes resulted in a range of 5–6 mm, across all datasets. The simulation developed herein results in an accuracy comparable to others in the literature [6], has real-time performance, required less data preparation (as a consequence of using a position-based dynamics approach) and made use of a larger dataset of porcine subject scans. Furthermore, the feasibility of modelling pneumoperitoneum in humans was initiated. A comparison between the biomechanical model used against those found in the literature is not straightforward as the model parameters involved are not directly comparable [9]. The simulation proposed herein is not necessarily superior when compared to the methods and respective results of those found in the literature; it rather aims at tackling a different problem, that of enabling patient-specific simulation to be more easily translated into clinical practice. Measurements of time taken to generate the porcine models are challenging as the amount of datasets to use was limited and the time to segment the regions of interest shortened with experience. However, once these structures have been segmented, generating surface and volumetric meshes for a stable position-based dynamics model is almost zero.

This study has some limitations. As the datasets from group #1 were acquired at an external facility by a separate team, the settings used under CT acquisition were approximate. Such required the use of the dataset from group #2, which comprised only one subject, and the respective scans lacked contrast quality. The quantity of data processing required to generate the simulation model from the CT volumes might have induced a loss in precision due to error propagation. As the imaging data were not in the standards of typically acquired imaging for patients preoperatively, the data preparation was time-consuming. The error metrics used in the calibration and validation steps were generated accounting solely for the inflatable structure. A more robust simulation, considering the viscera and abdominal wall in the optimisation and validation processes, could have derived more precise results. The low precision in defining the landmarks in the human feasibility study may have reduced the accuracy of the findings. Furthermore, measurements on the surface of the abdominal wall might not be sufficient to reflect organ deformation and displacement. Only one dataset and respective measurements of humans was possible due to the limited number of patients listed for surgery within the time period. Even though porcine anatomy is a good surrogate of human anatomy, the existing discrepancy might have reduced accuracy. The simulation pressure parameter used to simulate inflatables within the FleX framework is in practice a volume variation parameter. Manipulating volume has a different biomechanical impact when compared to simulating pressure. However, simulating pressure as a variation in volume was considered a valid approximation, as results demonstrated a level of accuracy similar to that found in the literature and with the additional benefit of enabling a more straightforward data preparation. The latter is required when simulating patient-specific models in real time. Regarding the implementation of the simulation, both the soft bodies (abdominal wall and viscera) were defined with the same cluster stiffness coefficient, when ideally one cluster stiffness coefficient should be defined per soft body. Using the same cluster stiffness coefficient simplified the optimisation process and assured that the parameters would not become over-fitted. This way, for all abdominal organs, using a constant cluster stiffness coefficient provided enough accuracy when compared to that found in the literature.

The feasibility of modelling pneumoperitoneum in humans was demonstrated. However, a robust validation of translating this simulation to humans should account for more measurements across a higher amount of patients. The Kinect, optical trackers or laser scan would be feasible techniques to provide a higher level of accuracy when measuring variations due to pneumoperitoneum of patients intraoperatively [1, 2]. Modelling a specific organ displacement or deformation due to pneumoperitoneum could be possible with an approach similar to that presented in Johnsen et al. [10], where the influence of pneumoperitoneum in the shape and location of the liver was assessed. The implementation adopted herein facilitates a real-time visualisation and interaction with the model, and as such, this framework could assist the surgeon in surgical planning, specifically in rehearsing and defining the optimal surgical strategy in a patient-specific manner. An algorithm could be developed to estimate the precise port positions automatically, knowing the exact FOV and ergonomics desired by the surgeon. A translation into clinical practice, by means of AR, would enable an overlay of pneumoperitoneum model onto the patient, which could facilitate translating the estimation of trocar positioning onto the patient. Furthermore, the entire simulation framework could be integrated into a virtual reality surgical simulator to allow a more realistic understanding of the intraoperative scenario, i.e. a system that proposes port positions which the surgeon can evaluate or adjust as needed before making any incisions.

## Footnotes

1 Data sourced at the courtesy of Matt Clarkson, Centre for Medical Image Computing (CMIC), UCL.

2 Data were used for the work developed by Johnsen et al.

3 F.A.S.T.^®^ Interactive Segmentation powered by Fovia’s *High Definition Volume Rendering*^®^*Software*.
